# Heart rate modulation in stable coronary artery disease without clinical heart failure: What we have already learned from SIGNIFY?

**DOI:** 10.1016/j.conctc.2016.06.003

**Published:** 2016-06-23

**Authors:** Gian Piero Perna, Fabio Vagnarelli, Maurizio Volterrani, Ilaria Battistoni, Claudio Rapezzi

**Affiliations:** a Cardiology, Cardiovascular Department, “Ospedali Riuniti di Ancona”, Ancona, Italy; b Cardiology, Department of Experimental Diagnostic and Specialty Medicine, Alma Mater Studiorum-University of Bologna, Bologna, Italy; c Cardiology, “San Raffaele Hospital”, Roma, Italy

## Abstract

An elevated heart rate is a marker of cardiovascular risk in patients with stable coronary artery disease. Ivabradine selectively inhibits the “*f*” current in the sinus node and reduces heart rate without any modifications of blood pressure, myocardial contractility and arteriolar resistance. However the addition of ivabradine to standard therapy to reduce heart rate did not improve outcomes in the recent SIGNIFY trial. Moreover, a significant interaction between the effect of ivabradine among subgroups with and without angina was detected, with a worse outcome in patients in CCS class >II at baseline. The explanation for this surprising finding despite a significant reduction in angina and myocardial revascularization procedures is uncertain. A J-curve for heart rate was not demonstrated. We speculate a significant interference on adverse events (mainly atrial fibrillation and consequently acute coronary syndromes) and on the outcome of unfavorable interactions between ivabradine and diltiazem, verapamil and strong inhibitors of CYP3A4 (4.6% of the total population).

Indeed, when these patients are excluded from subgroup analysis, the harmful effect of Ivabradine among patients with severe angina disappears.

In conclusion, heart rate is a marker of risk but is not a risk factor and/or a target of therapy in patients with stable coronary artery disease and preserved ventricular systolic function. Standard doses of ivabradine are indicated for treatment of angina as an alternative or in addition to beta-blockers, but should not be administered in association with CYP3A4 inhibitors or heart rate-lowering calcium-channel blockers.

## 1 Introduction

A high resting heart rate (HR) is a marker of risk in patients with heart failure [1], asymptomatic left ventricular systolic dysfunction [2], stable coronary artery disease [3], and in subjects with cardiovascular risk factors [4]. Indeed, an increase of HR determines several pathophysiological changes leading to adverse cardiac events: endothelial dysfunction and increase of oxidative stress, plaque instability, increased myocardial oxygen consumption, reduction of diastole duration with consequent reduction of coronary perfusion, remodeling and hypertrophy of left ventricle, reduction of left ventricular filling duration, and decrease of myocardial contractility [1], [2], [3], [4], [5]. By counteracting these unfavorable mechanisms, HR pharmacological modulation may improve symptoms and outcome.

Ivabradine selectively inhibits the “*f*” current in the sinus node [6] and reduces the heart rate in a “pure” way, without any modifications of blood pressure, myocardial contractility and arteriolar resistance. Because of these pharmacological properties, implementation of ivabradine to standard therapy was tested in several randomized trials to assess its effects through the cardiovascular continuum.

In chronic heart failure (HF) secondary to left ventricular systolic dysfunction (LVSD), the addition of ivabradine to standard therapy improves symptoms and outcome: in SHIFT study primary composite endpoint (cardiovascular death or hospital admission for worsening heart failure) was reduced by 18% compared to placebo (NNT 24). These effects were strictly related to baseline (pre-treatment) heart rate and to the extent of heart rate reduction after 4 weeks of therapy [1], [5], [7], [8].

In the setting of coronary artery disease (CAD) with asymptomatic LVSD and HR > 70 bpm, ivabradine reduces the rate of hospitalization for fatal and non fatal MI, particularly in patients suffering from angina [9].

In patients with symptomatic CAD and preserved left ventricular function (PLVEF), ivabradine proved to be as effective as beta-blockers to achieve adequate control of angina(10). Moreover, ivabradine in combination with beta-blockers has shown a superior anti-anginal and anti-ischemic effect during stress test, compared to beta-blockers alone [11].

However, it was unknown whether reducing HR by ivabradine on top of standard therapy improves outcome of patients with stable CAD and PLVEF [12], [13]. Therefore, the SIGNIFY study was designed and performed to test this hypothesis [14].

## 2 Rationale and methodology

The SIGNIFY study was conducted to verify a very ambitious hypothesis: reducing cardiovascular mortality and MI (fatal or not) of patients with stable CAD and without clinical HF, through a pure HR modulation with Ivabradine on top of current standard therapy (including statins, antiplatelet drugs, ACE-inhibitors, beta-blockers).

The research hypothesis was that lowering HR below 60 bpm could reduce myocardial ischemia, myocardial oxygen consumption, endothelial dysfunction, and oxidative stress.

A rigorous methodology was used by investigators to reach this ambitious goal: 19,102 patients aged >55 years, who had both stable CAD without clinical HF and a HR of 70 bpm or more, were randomly assigned to placebo or ivabradine, at a dose of 7.5 mg up to 10 mg twice daily, with the dose adjusted to achieve a target HR of 55–60 beats per minute. Participants had to be in sinus rhythm, have a resting heart rate of 70 beats per minute or more on two consecutive electrocardiograms (during “run-in” period), and have documented CAD or myocardial ischemia. Patients with left ventricular dysfunction (left ventricular ejection fraction ≤40%) were excluded.

Patients aged> 75 years were treated with 5 mg twice daily and this dose was also used in case of excessive HR reduction with the high dose.

The study included 12,049 patients (63%) with activity-limiting angina [class ≥II on the Canadian Cardiovascular Society scale). This was a prespecified group in which the extent of the symptoms did suggest a worse prognosis.

The primary end point was a composite of death from cardiovascular causes or nonfatal myocardial infarction. The secondary end points included the components of the primary end point (death from cardiovascular causes and nonfatal myocardial infarction) as well as death from any cause.

The mean age of the study population was 65 years, 72.4% of the patients were men, and the mean resting HR was 77.2 beats per minute. There was no evidence of left ventricular systolic dysfunction in the overall study population (mean ejection fraction, 56.4%).

Most patients were receiving appropriate therapy for cardiovascular disease (beta-blockers in 83.1%, statins in 92.2%, antiplatelet therapy or anticoagulants in 97.7% of the patients, and ACE inhibitors in 59.3%)

According to the study protocol, 51% of patients were treated with ivabradine 10 mg twice daily, 27% with 7.5 mg twice daily, 22% with 5 mg twice daily.

## 3 Main findings

Ivabradine reduced mean HR by 10 bpm compared to placebo (at 3 months 60.7 ± 9.0 beats per minute with ivabradine and 70.6 ± 10.1 beats per minute with placebo). The difference in the mean heart rate between the ivabradine group and the placebo group was maintained for the duration of the study in the total population and in the subgroup of patients with activity-limiting angina at baseline. 30% of patients treated with ivabradine reached a heart rate <50 bpm.

The rates of permanent discontinuation of the study drug were 20.6% in the ivabradine group and 14.5% in the placebo group (p < 0.001).

The main reason for study-drug withdrawal in the ivabradine group was asymptomatic bradycardia and, to a lesser extent, symptomatic bradycardia. A higher incidence of bradycardia-dependent atrial fibrillation was found in the ivabradine group compared to placebo (5.3 vs. 3.8%; p < 0.01).

**Fig. 1:**
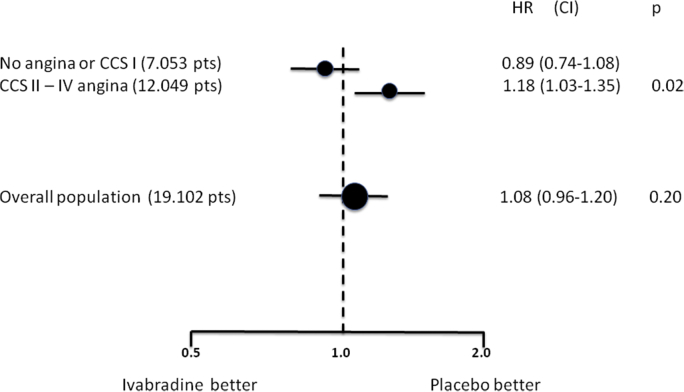
Hazard ratios and 95% confidence interval for the primary endpoint (composite of death from cardiovascular causes or nonfatal MI) in the overall population and in prespecified angina subgroups in SIGNIFY trial.

There was no significant difference in the incidence of the primary end point (composite of death from cardiovascular causes or nonfatal MI) between the ivabradine group and the placebo group (6.8% and 6.4%, respectively; hazard ratio, 1.08; 95% confidence interval [CI], 0.96 to 1.20; P = 0.20; Fig. 1). Moreover, there were no significant between-group differences in any other secondary end points.

Subgroup analysis showed only one significant interaction (P = 0.02) between the study treatment and the presence of angina at baseline in the prespecified subgroup defined according to CCS class (P = 0.02), with opposite findings among subgroups of patients without angina (or CCS I) and those with CCS II–IV angina (Fig. 1). Ivabradine was associated with a modest albeit significant increase in the incidence of the primary end point among patients who had angina of CCS class II or higher (7.6%, vs. 6.5% with placebo; hazard ratio, 1.18; 95% CI, 1.03 to 1.35; P = 0.02) but not among patients without angina or those who had angina of class I (hazard ratio, 0.89; 95% CI, 0.74 to 1.08; P = 0.25).

**Fig. 2:**
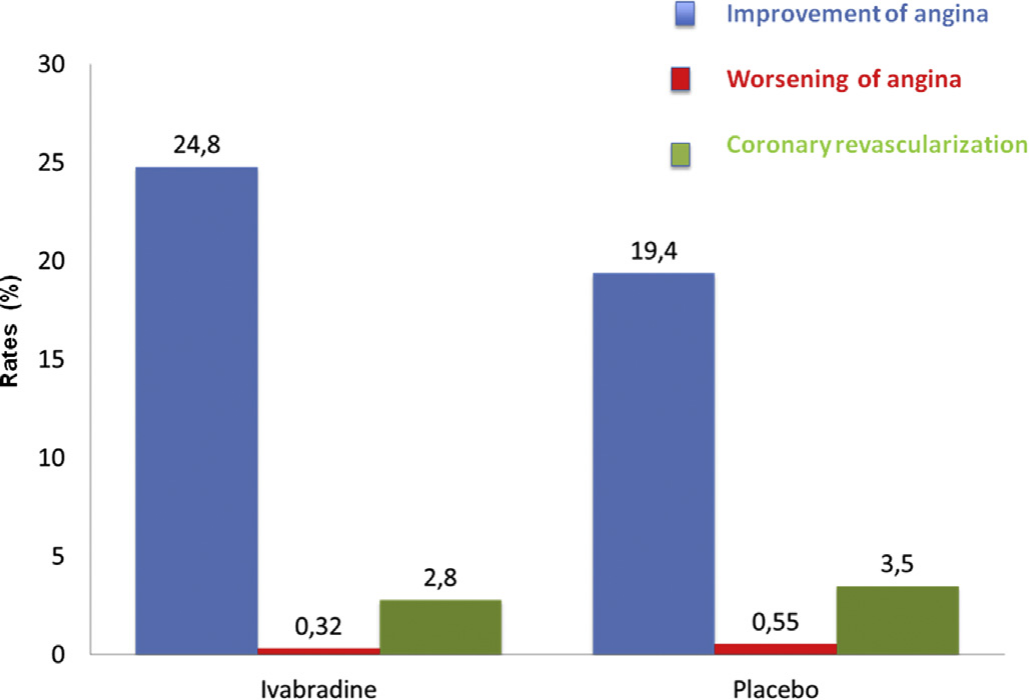
Effects of Ivabradine vs. Placebo on angina control and coronary revascularization.

In contrast to the distribution of coronary events, a significant improvement of angina (24.8% in ivabradine, 19.4% in placebo, Δ = 5.4%; p < 0.01) and a significant reduction of elective myocardial revascularization (ivabradine 2.8%, placebo 3.5%, Δ = 0.7%, HR 0.82; p < 0.01) were found in the subgroup with CCS II–IV angina (Fig. 2). In the subgroup of patients with CCS II–IV angina treated with ivabradine, the main determinant of the negative outcome was an increase of non-fatal MI (HR 1.18, 95% CI 0.97–1.42; p = 0.09).

## 4 Interpretation of results

The SIGNIFY trial provides several pathophysiological implications.

Firstly, the results of the study clearly show that HR is a risk marker but not a risk factor in patients with CAD and PLVEF receiving appropriate background therapy (ACE inhibitors, statins and antiplatelet agents platelet). HR reduction in a population like that, who is “a priori” at relatively low risk (2.8%/year) thanks to current standard treatment, did not increase plaque stability; thus, the hypothesis that HR itself may determine coronary events was denied in the contemporary era of extensive antithrombotic/statin therapy.

Secondly, the hypothesis that reducing ischemia and angina may improve the prognosis was gainsaid too; actually, this result is not a complete surprise. In patients with stable CAD and normal ejection fraction, no drug among those used for symptom relief (including beta-blocker) has proved to ameliorate prognosis [15]. Indeed, in current European guidelines these drugs are consistently regarded as treatments aimed at improving symptoms, not prognosis [16]. Even myocardial revascularization with angioplasty, which represents the most effective treatment for angina [17], was not able to improve outcome in COURAGE study [18], except for patients with severe angina and/or ischemia.

**Fig. 3:**
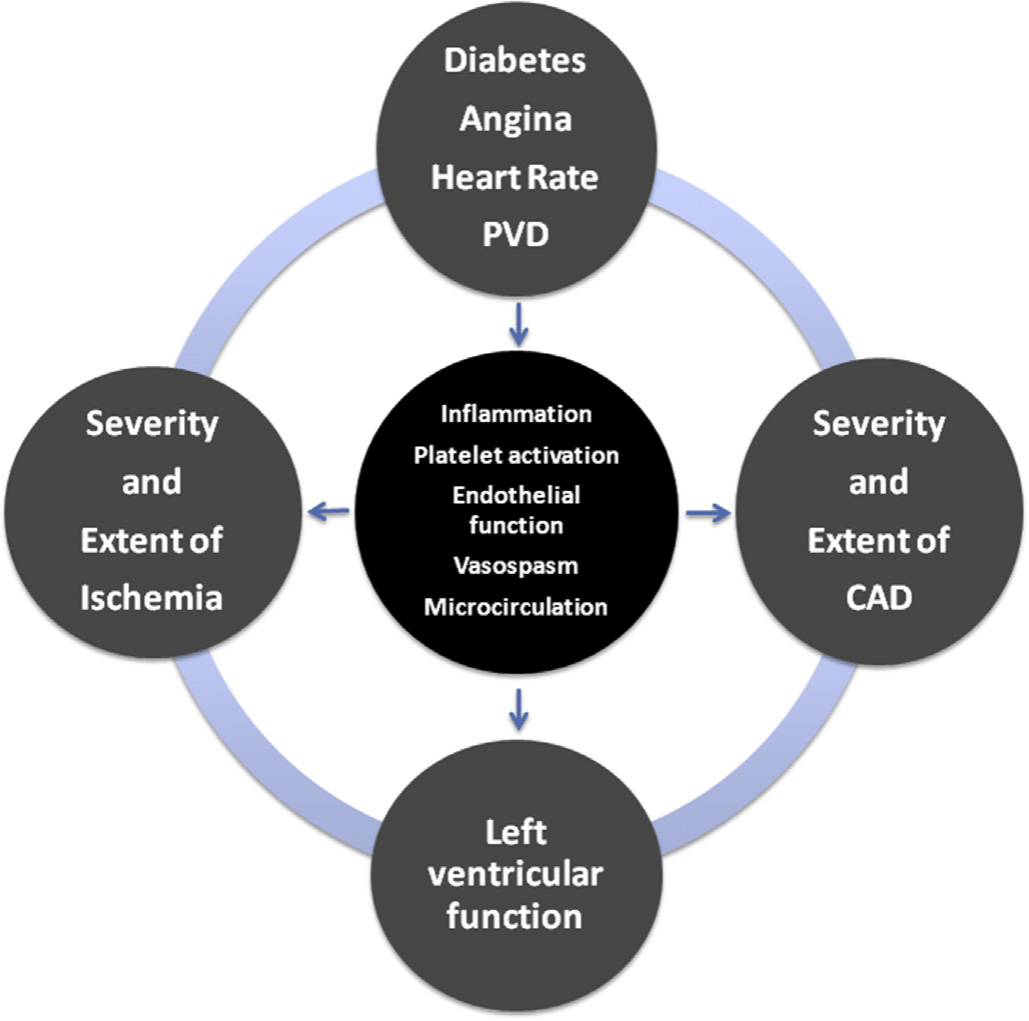
Determinants of prognosis in stable Coronary Artery Disease: a complex interplay between clinical, anatomical, functional, and pathophysiological factors.

Indeed, prognosis of patients with stable CAD is affected by several factors (Fig. 3); the complex interplay between ischemia, coronary vessel disease, functional components, and clinical parameters implies that a single pharmacologic intervention has little chance to be effective, unless it acts on factors with a pivotal role in the cascade leading to coronary events, such as plaque stability (statins) or thrombosis (antiplatelets drugs).

However, the crucial “issue” within SIGNIFY study remains the highest frequency of adverse events, mainly non fatal MI, in the subgroup of patients with severe angina; among these, ivabradine treatment was associated with a reduction of revascularization and a substantial improvement of symptoms, but also with a worse outcome, whereas ivabradine determined a non significant trend towards better outcome in patients without angina.

The explanation of this surprising finding is uncertain, although it should be treated with caution since the results of the primary efficacy analysis were not significant. Whenever, in clinical trials, opposite effects occur in subgroups of patients, it is also mandatory to look for possible mechanisms underlying them. Indeed, they may also be “spurious” or the result of chance. A possible mechanism could stem from excessive bradycardia with subsequent reduction of coronary perfusion in patients with a greater extent of coronary artery lesions.

**Fig. 4:**
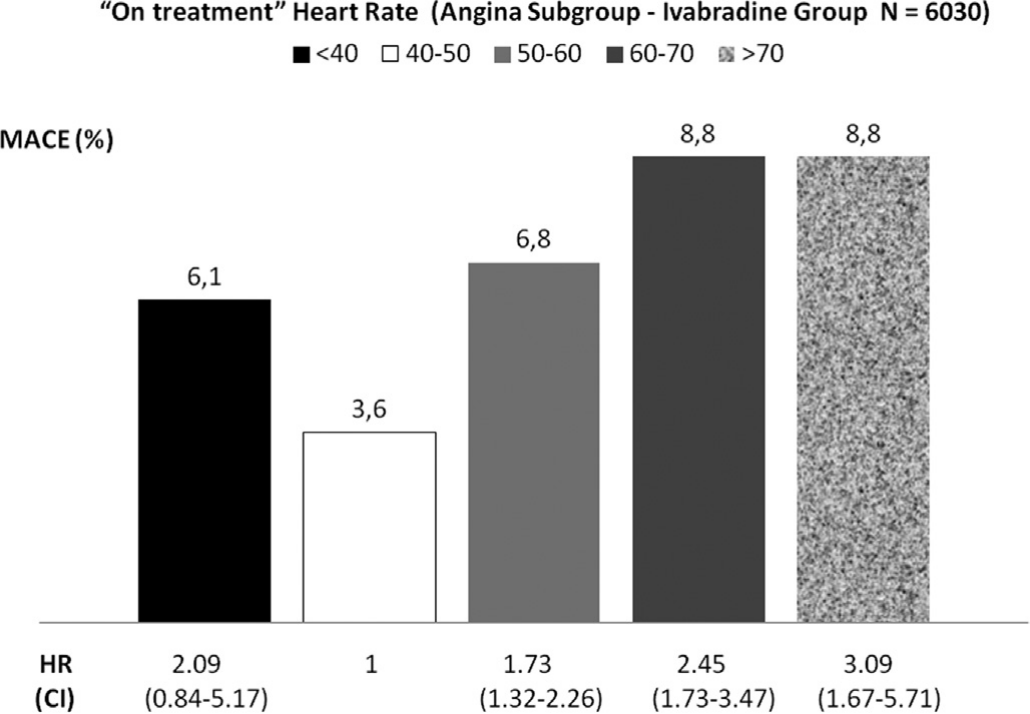
Relationship between “on treatment” heart rate and major adverse cardiac events (MACE) in Angina patients (Ivabradine Group). HR: hazard ratio. CI: confidence interval.

However findings from an analysis performed by the Pharmacovigilance Risk Assessment Committee of European Medicines Agency (EMA) exploring the relationship between “on treatment” Heart rate and outcome are against this hypothesis [19]. The main results of EMA post-hoc analysis focused on patients with angina and treated with ivabradine are summarized in Fig. 4: the lowest number of MACE occurred in patients with heart rate 40–50 bpm, the highest frequency of MACE was found among patients with HR > 70 bpm, whereas a slight but not statistically significant increase of MACE was detected in patients with HR < 40 bpm. In addition Cox-regression analysis did not show an excess risk associated with lower HR. Thus, the lack of a “J curve” and of a close relationship between the primary end-point and HR suggest that other mechanism may be operating [14], [19].

**Fig. 5:**
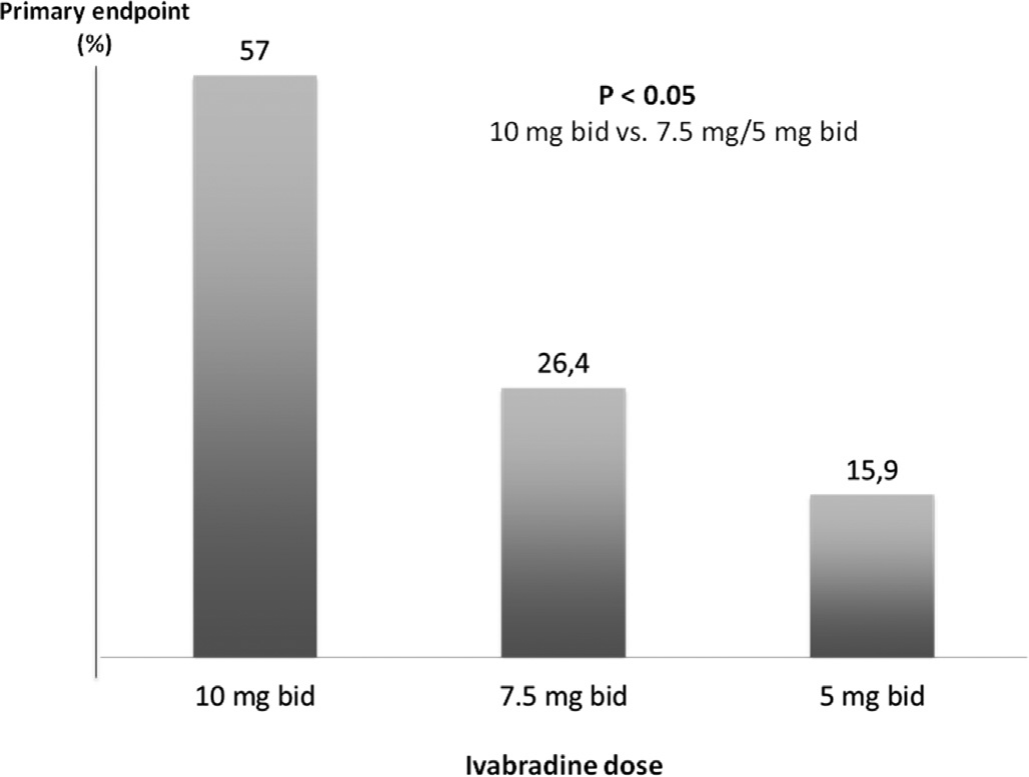
Distribution of primary endpoint occurrence according to Ivabradine dose. The 10 mg bid dose is not approved by European Medicines Agency (EMA).

The use of ivabradine “unusual” doses (51% of patients received 10 mg twice daily) has certainly contributed to the development of side effects, which were significantly higher in SIGNIFY than any other clinical trials with ivabradine. Accordingly, most primary endpoints occurred at the highest dose (57.7%) compared to the 7.5 mg (26.4%) and 5 mg (15.9%), with similar results for the individual endpoints (Fig. 5).

**Fig. 6:**
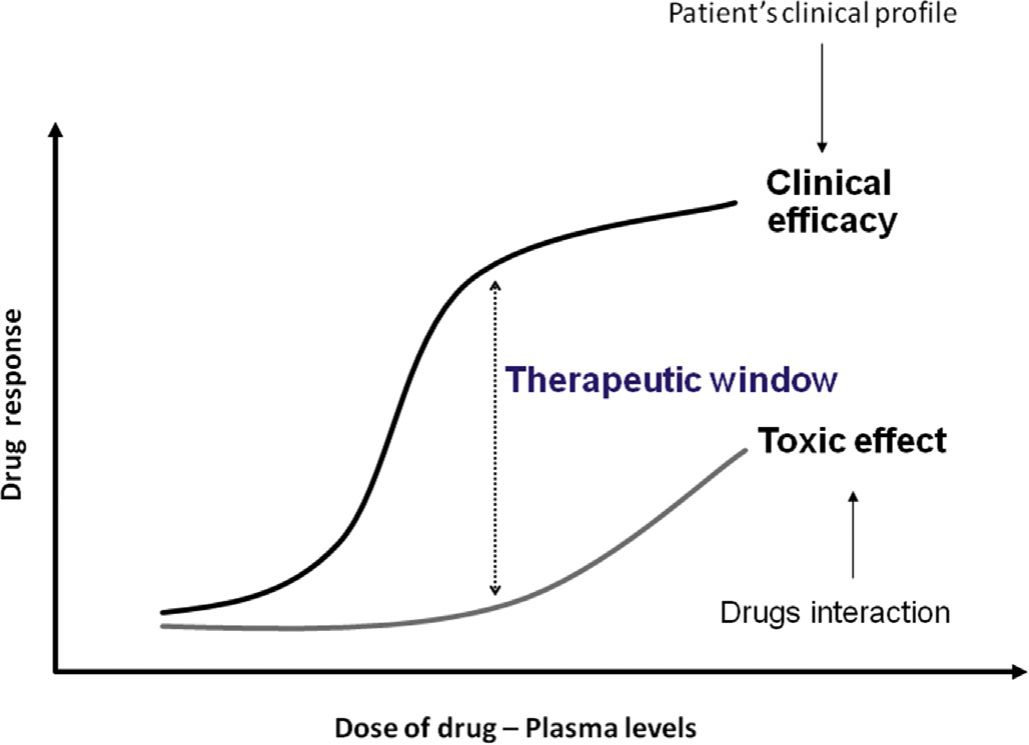
Theoretical relationship between the dose of a drug and the response (in terms of clinical efficacy and toxic effect).

For all drugs, increasing the dose increases the likelihood of side effects, proportionally to the plasma levels of the drug itself (Fig. 6).

**Fig. 7:**
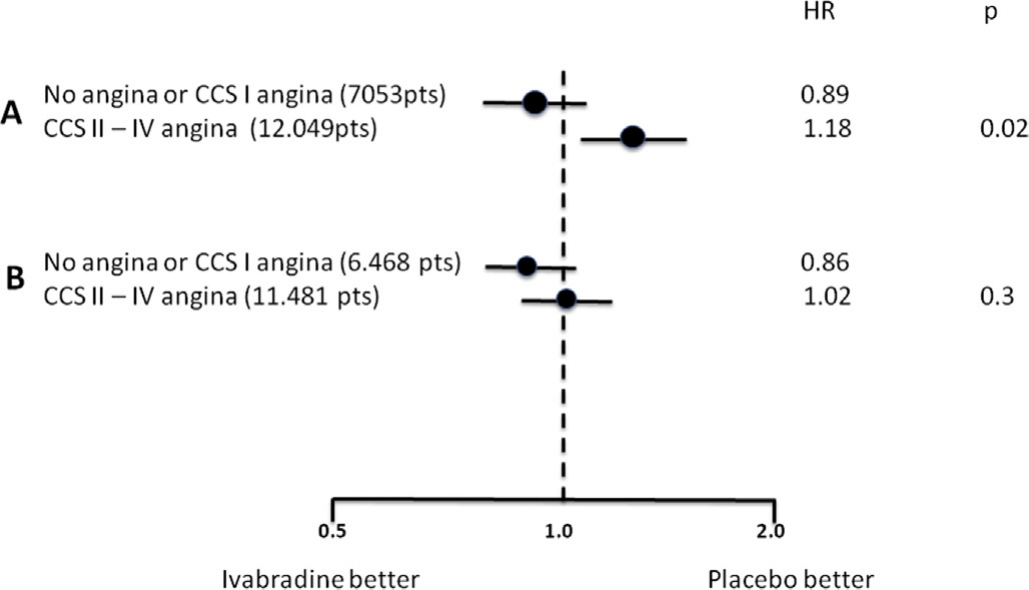
Primary end-point occurrence in angina subgroup analysis of SIGNIFY Trial. A: When the overall population is considered, a statistically significant interaction is detected between subgroups with or without severe angina. B: When patients receiving Diltiazem/Verapamil (1135) or strong CYP3A4 inhibitors (262) are excluded from analysis, the difference between groups and the harmful effect of Ivabradine among patients with severe angina disappear.

In addition, other mechanisms seem to have contributed to an unpredictable increase of ivabradine plasma levels and consequently to side effects. Within SIGNIFY study population, 1135 enrolled patients were treated also with diltiazem or verapamil, and 262 were taking strong CYP3A4 inhibitors, which increase ivabradine plasma levels. In the overall study population, this pharmacological interactions were associated with a worse outcome, both for the primary endpoint (risk increased by 43%, interaction p-value = 0.062) and for non-fatal MI (risk increased by 64%, interaction p-value = 0.006). As mentioned above, when the overall population of SIGNIFY trial is considered, a statistically significant interaction is detected between subgroups with or without severe angina. However, when patients receiving Diltiazem/Verapamil or strong CYP3A4 inhibitors are excluded from analysis, the difference between groups (with or without severe angina) and the harmful effect of Ivabradine among patients with severe angina disappear (Fig. 7). The increased frequency of nonfatal MI, which is the main “driver” of the unfavorable effect found in ischemic subgroup, can be partially explained by the higher frequency of atrial fibrillation (+1.5%, p < 0.001) in the ivabradine group [20], due to a bradycardia-dependent mechanism (in particular for patients treated with verapamil/diltiazem, or CYP3A4 inhibitors). Indeed, atrial fibrillation, when occurring in patients with angina (CCS II–IV) and severe CAD, may easily lead to chest pain, ECG abnormalities and/or troponin increase with subsequent non fatal MI.

## 5 Clinical implications

SIGNIFY may be considered a pathophysiological study, conducted with a “strict” methodology, according to which more than half patients in the ivabradine group (51%) received doses not approved for clinical use by regulatory agencies. In addition, a small but not negligible subgroup of patients (4%) was treated with drugs not recommended to be used in combination with ivabradine (among these the frequency of adverse events doubled). Lastly, the management of patients suffering from severe angina (CCS> II) with medical therapy alone does not reflect current clinical practice; in this clinical context, ESC guidelines recommend coronary angiography and myocardial revascularization to improve symptoms [15]. Similarly to other studies conducted with a methodology which is not usual in clinical practice, it is difficult to draw conclusions that are valid for the clinician.

However, SIGNIFY study may provide some clinical implications:

1 HR modulation with ivabradine has no prognostic impact but it may be useful for symptoms relief, as already shown by previous studies [10], [11]. ESC guidelines on stable CAD recommend ivabradine for this purpose indeed [15].
2 In patients with stable angina, ivabradine should be used as an alternative to beta-blockers (if contraindicated or not tolerated) or in combination with them. The recommended starting dose is 5 mg twice daily and may be up-titrated to 7.5 mg tid or down-titrated to 2.5 mg twice daily, if necessary. A starting dose of 2.5 mg twice daily may be recommended for older patients [1], [19]. As HR modulation in stable CAD provides no clinical benefit, it is not necessary to reach a definite HR target.
3. When using ivabradine, attention should be paid to possible drug interactions, and associations with diltiazem, verapamil or strong inhibitors of CYP3A4 must be absolutely avoided.
4. Clinical examination at 3 months in patients receiving ivabradine is useful for checking the effectiveness of treatment on symptoms and its tolerance.

After SIGNIFY was published, regulatory authorities started a procedure to assess the safety of ivabradine in patients with stable CAD. However, there is a lot of data on the safety of the drug when used in standard doses according to the indication approved by regulatory authorities, derived from several clinical trials. Indeed, these evaluated the safety of Ivabradine in patients with stable angina, in patients with ischemic heart disease and left ventricular dysfunction [3], [9], and in patients with heart failure and severe LVD [1], [4], [5], [7].

Moreover, important insights about the efficacy and safety of ivabradine are provided by AIFA (Agenzia Italiana del Farmaco) real-world registry, collecting data from all online drugs prescriptions made by Italian Cardiologists. From February 2008 to October 2009, 14,256 patients were included. In this population, the rates of serious adverse events were extremely low (0.4%), with a rate of minor side effects (mainly phosphenes) lower than 1% and an extremely low percentage of cardiovascular death (16 patients, <0.1%). On the other hand, treatment with Ivabradine provided excellent symptoms relief (angina reduced by 80% during observation period).

## 6 Conclusions

Despite a high HR being a risk marker in patients with stable CAD and preserved left ventricular function, the pharmacological modulation of heart rate obtained by adding ivabradine to standard therapy does not provide significant prognostic benefits [14]. Given that primary cardiovascular effect of Ivabradine is to reduce HR, SIGNIFY suggests that an elevated HR is only a marker of risk – but not a modifiable determinant of outcome-in patients who have stable CAD without clinical HF.

Even in patients with CCS II–IV angina, the clinical benefit of Ivabradine is limited to symptoms relief and to reduced need for revascularization, without any effect on prognosis; therefore, reducing angina does not imply better outcome. These results are not surprising: in stable CAD with preserved left ventricular function, all strategies aimed at symptoms relief (including PCI) did not improve prognosis when added “on top” of secondary prevention therapy (statins, antiplatelet agents, and ACE-inhibitors) [16], [18].

Similarly to other clinical trials aimed at maximizing pharmacological intervention, findings from SIGNIFY confirms that “safety” may be hampered by drug interactions and by unusual doses with subsequent unexpected and confounding results [21], [22], [23].

The results of Signify will not modify the management of stable angina in daily clinical practice or guidelines on this topic. In case of preserved left ventricular function, ivabradine remains a “second-line” drug for symptoms control, in addition to beta-blockers or alternatively to them. In this context, coronary revascularization should be considered only when medical therapy is ineffective or partially effective; however, clinical efficacy should not be judged on the basis of the achieved HR. In other words, there’s not enough evidence for a strict HR target to reach in stable CAD, even if SIGNIFY study does not denies in fact US guidelines recommendation of a HR target between 55 and 60 bpm [12], [15].

In case of HF with severe LV dysfunction, when HR is still above 70 bpm, the combination of ivabradine with beta-blockers remains strongly recommended, not only for symptoms relief, but to improve prognosis [9], [15].

After SIGNIFY it would be desirable to verify the effectiveness of all drugs used in the treatment of stable angina without LV dysfunction through prospective studies of appropriate size.

## Conflict of interest

No conflict of interests to be disclosed.
